# Development and Validation of a Mediterranean Oriented Culture-Specific Semi-Quantitative Food Frequency Questionnaire

**DOI:** 10.3390/nu8090522

**Published:** 2016-08-25

**Authors:** Elpiniki Athanasiadou, Charikleia Kyrkou, Maria Fotiou, Foteini Tsakoumaki, Aristea Dimitropoulou, Eleni Polychroniadou, Georgios Menexes, Apostolos P. Athanasiadis, Costas G. Biliaderis, Alexandra-Maria Michaelidou

**Affiliations:** 1 1st Department of Obstetrics and Gynecology, School of Medicine, Aristotle University of Thessaloniki, Thessaloniki 541 24, Greece; athanasiadou.niki@gmail.com (E.A.); apostolos3435@gmail.com (A.P.A.); 2 Department of Food Science and Technology, School of Agriculture, Faculty of Agriculture, Forestry and Natural Environment, Aristotle University of Thessaloniki, Thessaloniki 541 24, Greece; ckyrkou@hotmail.gr (C.K.); fotioum@yahoo.gr (M.F.); foteinitsak@hotmail.com (F.T.); dimitropa@hotmail.com (A.D.); elpol@ee.auth.gr (E.P.); biliader@agro.auth.gr (C.G.B.); 3 Department of Field Crops and Ecology, School of Agriculture, Faculty of Agriculture, Forestry and Natural Environment, Aristotle University of Thessaloniki, Thessaloniki 541 24, Greece; gmenexes@agro.auth.gr

**Keywords:** Mediterranean diet, culture, nutritional assessment, food frequency questionnaire, validation, nutrients, Bland-Altman, cross-classification, pregnancy

## Abstract

The objectives were to develop a Mediterranean oriented semi-quantitative food frequency questionnaire (FFQ) and evaluate its validity in measuring energy and nutrient intakes. For FFQ development, the main challenge was to merge food items and practices reflecting cultural Mediterranean preferences with other food choices ensuing from diet transition to more westernized dietary patterns. FFQ validity was evaluated by comparing nutrient intakes against the average of two 24-h dietary recalls for 179 pregnant women. Although the mean intake values for most nutrients and energy tended to be higher when determined by the FFQ, the Cohen’s d was below 0.3. Bland-Altman plots confirmed the agreement between the two methods. Positive significant correlations ranged from 0.35 to 0.77. The proportion of women classified correctly was between 73.2% and 92.2%, whereas gross misclassification was low. Weighted kappa values were between 0.31 and 0.78, while intraclass correlation coefficients were between 0.49 and 0.89. Our methodological approach for the development and validation of this FFQ provides reliable measurements of energy, macro- and micronutrient intakes. Overall, our culture-specific FFQ could serve as a useful assessment tool in studies aiming at monitoring dietary intakes, especially in the Mediterranean region, where countries share common cultural dietary habits.

## 1. Introduction

Over the last few decades, there has been a steady increase in epidemiological studies that provide fundamental insights into the dynamic relationship between diet and health [1]. In this context, the current momentum towards the Mediterranean diet has solid biological foundation and does not represent a transient fashion, since literature is characterized by burgeoning interest in examining the health benefits of this dietary pattern [2,3]. The Mediterranean diet is a multifaceted nutritional approach, since it involves not only dietary aspects but also lifestyle, socio-cultural, and environmental dimensions [4]. However, for many countries in the Mediterranean area, several lines of scientific evidence indicate a shift away from the essential rules of the traditional eating patterns and point towards a tendency to adopt a more westernized dietary model [5,6,7,8,9,10,11].

In order to evaluate dietary habits, different methods have been employed. Food-frequency questionnaires (FFQs) have been shown to be valuable tools for evaluating habitual dietary intake [12,13,14], since they offer, with a single measurement, a better approximation of the habitual diet over a specific period, in comparison with short term food records that capture only a snapshot of a few days’ diet [12,15]. The ease of administration and the relative low cost of implementation facilitate further data collection [16,17]. A common approach, in epidemiological studies, is to adapt previously developed FFQs [18,19]. However, the development and use of cultural specific FFQs, entirely based on dietary practices of different ethnic populations, is preferable and highly needed, due to culturally unique foods, varying cultural definitions of food groups and serving sizes, as well as bridging traditional dietary habits with current dietary trends [11,20,21]. It is worth mentioning that before implementation, culture-specific FFQs have to be validated, to define the degree of accuracy within the target population [12,13,22,23,24].

Pregnant women represent a target population group that attracts considerable scientific interest, since research findings diachronically advance our knowledge of the role of maternal nutrition on fetal growth, pregnancy outcome, and health of the offspring in adult life [25,26,27]. Within this frame, the health-promoting and preventive dimensions of Mediterranean diet during pregnancy have been recently acknowledged [11,28,29,30].

Considering the above facts, the objective of the present study was twofold: (a) to develop a Mediterranean oriented culture-specific semi-quantitative FFQ, with a priority challenge to bridge food items and practices reflecting cultural Mediterranean preferences with other food choices ensuing from diet transition to more westernized dietary patterns; and (b) to evaluate the relative validity of the developed FFQ in measuring energy and nutrient intakes, among a population of pregnant women. In a long-term perspective, this assessment tool will be used in our ongoing studies, focusing on the effect of nutrition—in terms of habitual diet—on pregnancy evolution and outcome.

## 2. Materials and Methods

**Figure 1:**
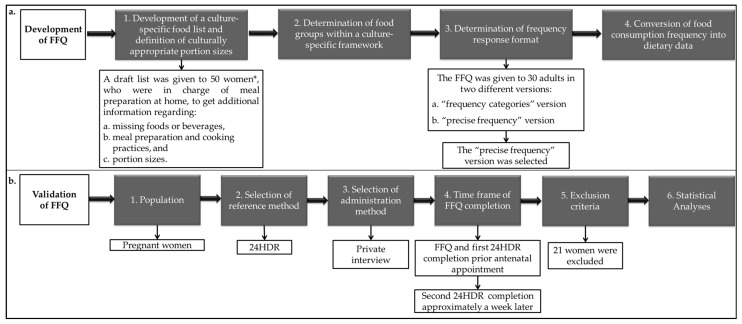
Methodological design for (**a**) the development and (**b**) validation of the present culture-specific food frequency questionnaire. FFQ: food-frequency questionnaire; 24HDR: 24-h dietary recall. * For details see Section 2.1.1.

The methodological design of the present study is schematically outlined in Figure 1.

### 2.1. FFQ Development

#### 2.1.1. Development of a Culture-Specific Food List and Definition of Culturally Appropriate Portion Sizes

To develop a culture-specific food list, a draft list, based on previous experience from small-scale surveys conducted by our research group, was utilized. This draft list was administered to 50 women who were in charge of meal preparation at home—irrespective of their employment status, education, or household income—with the aim to check its clarity and completeness. Participants could provide additional information on open-ended sections regarding: (a) missing foods or beverages; (b) meal preparation and cooking practices; and (c) portion sizes. In more detail, (a) foods and beverages reported from more than one respondent were included; (b) women were requested to describe the cooking method (roasted/grilled/baked, boiled, fried), as well as to report the amount of olive oil used during meal preparation; and (c) commonly used household utensils (such as spoons or plates) or “natural” units (e.g., one apple, egg, etc.) were used in order to capture the participant’s perception of portion size.

Based on the participants’ responses, it was decided that the FFQ matrix would be composed of 221 foods. The decision on olive oil addition to the different recipes was facilitated by the fact that cooking instructions in Greece are most frequently given in spoons or cups of specific dimensions. In an attempt to reach a consensus on portion size for each food, the differences in perception on portions among the 50 participants were discussed. This consensus was then taken into account in the design of the FFQ.

#### 2.1.2. Determination of Food Groups within a Culture-Specific Framework

In order to facilitate dietary reporting, the following food groups were created: (1) bread/non sweet bakery/breakfast cereals; (2) pies/pastries; (3) pasta/rice/potatoes/other traditional starchy dishes; (4) “ladera”; (5) pulses; (6) raw vegetable salads; (7) cooked vegetable salads; (8) fruit; (9) nuts; (10) milk/dairy products; (11) meat/traditional meat dishes; (12) meat products; (13) eggs; (14) fish/seafood; (15) fats/spreads; (16) traditional dips/sauces/dressings; (17) sugar/sugar preserves/confectionary; (18) “ready-to-eat” foods; (19) chips/salty puffed snacks; (20) teas/coffee; (21) nonalcoholic beverages; and (22) alcoholic drinks. Food items were grouped by taking into account not only their nutrient content, but also the advice and input of the 50 women—in charge of meal preparation—participating in the preliminary study. Therefore, to be in line with practices and preferences reflecting current dietary habits: (a) “ladera”, i.e., cooked seasonal vegetables, mostly in olive oil based sauce, combined with tomato, onions, garlic, and herbs, as well as pulses, were listed as main dishes; (b) traditional dishes like stuffed zucchini or eggplant with minced meat, as well as popular dishes like mousaka and stewed beef, were grouped into “meat/traditional meat dishes” category; and (c) “ready-to-eat” foods group—i.e., restaurant prepared foods—included not only traditional, but also other food choices ensuing from the diet transition to more westernized dietary patterns.

To facilitate even more the accurate description of dietary habits, subgroups were also introduced, e.g., the “fruit” group was subdivided into “fresh fruit”, “dried fruit”, and “homemade and processed juices”.

#### 2.1.3. Determination of Frequency Response Format

In order to decide on the proper frequency response format, the culture-specific FFQ was administered to 30 adults in two different versions: (a) “frequency categories” version, where participants were assigned a series of predetermined options most commonly referred in the literature [18,19] (“never or less than once per month”, “one to three times per month”, “once per week”, “two to four times per week”, “five to six times per week”, “once per day”, “two to three times per day”, “four to five times per day”, and “six or more times per day”) concerning food intake; and (b) “precise frequency” version, where each participant could provide, on open-ended questions, the personalized frequency of food intake. It is worth mentioning that in both versions, cross-check and summary questions on overall consumption of each food group were used to evaluate the consistency of answers. When the daily mean intakes of energy and nutrients were calculated for both frequency versions, it was noted that misestimating of nutrient intakes (either overestimation or underestimation) was likely to occur when using the “frequency categories” version, especially for particular food groups that predominate the Mediterranean diet. An example of overestimation is given below.

Example: Some participants reported eating a portion of raw salad or cooked vegetable salad every day and consuming a variety of vegetables, e.g., 2 times per week broccoli, 2 times per week cauliflower, 2 times per week tomato, and 1 time per week lettuce. In the “frequency categories” version, the only frequency type available on the FFQ for the salads consumed 2 times per week was the “2–4 times per week”, scored as 3. As such, the respondent was confronted as consuming 3 times per week each of these salads, i.e., the nutrient content of participant’s choices would be attributed to 10 portions per week, instead of 7. This fact led not only to an overestimation of the nutrients contained in the vegetables, but also to an overestimation of energy intake, mainly derived from the addition of olive oil. This observation on overestimation was further intensified, as the predetermined frequency options and olive oil addition applies also to other foods prevalent in the diet and served daily as side dishes (e.g., pasta, rice, potatoes).

The supportive example mentioned above was the stimulus to select the use of the “precise frequency” version, so as to improve the quality of the information gathered. Aiming at higher levels of reliability, this version has been, also, proposed by other researchers [11,13,14,31,32,33].

#### 2.1.4. Conversion of Food Consumption Frequency into Dietary Data

A Microsoft excel database was created in order to convert participants’ responses into dietary data. The 3-stage procedure was: (a) define the composition of all the food items included in the FFQ; (b) inform the excel database with the appropriate portion size by food; and (c) create the appropriate functions for the calculation of daily energy, nutrient intakes, and nutrient density (nutrient intake per energy intake), as well as the functions for the computation of dietary glycemic index (GI) and Mediterranean Diet Score (MDS) [7].

In more detail, (a) the nutrient composition of food items was derived from (1) the “Food Composition Tables and Composition of Greek Cooked Food and Dishes” [34]; (2) the food composition database developed at the Department of Social Medicine of the University of Crete [35]; and (3) the USDA food composition Database for Standard Reference, Release 26 [36]; (b) the database was informed with the appropriate portion size ensuing from the consensus of opinion among the 50 women participating in the FFQ development; and (c) the calculation of the daily dietary intake was accomplished following the steps shown in Table 1.

The holistic nutrient profile of each participant was, thus, computed, as soon as the reported frequencies were entered into the developed excel database.

### 2.2. FFQ Validation

#### 2.2.1. Population

Two hundred pregnant women were invited to participate in the FFQ validation study, while visiting the 1st Department of Obstetrics and Gynecology, Papageorgiou General Hospital, Thessaloniki, Greece, for routine antenatal appointment at 18–26 weeks of gestation. The vast majority of the participants were of Greek ethnic origin. All subjects gave their informed consent for inclusion before they participated in the study. The procedures followed were in accordance with the ethical approval attained by the Bioethics Committee of the Medical School, Aristotle University, Thessaloniki, Greece (A19479—26/2/08).

All women were asked to provide information on age, pre-gestational weight, height, smoking behavior, occupation, and level of physical activity. Pre-gestational body mass index (BMI) was calculated as weight (in kilograms) divided by standing height (in meters) squared, and BMI classification was assigned according to the World Health Organization (WHO) BMI criteria [39]. Smokers were defined as those participants who reported smoking at least one cigarette per day, while the rest of the participants were characterized as non-smokers. Educational level was recorded as the number of completed years of education (9, 12, >12 years). The short version of the International Physical Activity Questionnaire (IPAQ) [40] was used for the evaluation of the physical activity status. It was decided to use the IPAQ, rather than the Pregnancy Physical Activity Questionnaire (PPAQ) [41], since IPAQ has already been translated into Greek [42].

#### 2.2.2. Selection of Reference Method

The selection of the appropriate reference method is a vital component of a validation study. One of the most common reference methods is the 24-h dietary recall (24HDR) [11,16,17,33,43,44,45,46,47,48,49,50,51,52,53,54,55,56], due to numerous advantages. The 24HDR is a high-speed, less expensive method and has relatively low respondent burden [13,33,47,57]. Furthermore, compared to food diaries and weighted records, the 24HDR interferes less with the subject’s everyday habits, since the possibility of distortion the actual diet is minimized [13,47,58]. For the above-mentioned reasons, the 24HDR was selected as the reference method. 

#### 2.2.3. Selection of Administration Method

It is reported in the literature that, if practical, interviewer-administered FFQs should be used in preference to self-administered FFQs, since greater accuracy, quality, and adequacy of data collection is achieved [13,14]. In the present study, dietary recording (FFQ and 24HDRs) was accomplished via private interview with a registered dietician or a well-trained interviewer (food scientist/nutritionist).

#### 2.2.4. Time Frame of FFQ Completion

The period chosen for representation or “image” of food consumption (testing period), within our designed framework, was the “past month”. According to Baer et al., (2005) [45], the assessment of diet during the past month is expected to be more accurate for pregnant women, since they may change their dietary habits within each trimester due to several factors, such as appetite, psychological state, nausea or vomiting [59]. This specific period was also adopted in numerous studies concerning pregnancy [29,45,46,51,60,61,62,63,64]. The testing period is a key factor for the number of recall days required [12]; therefore, two 24HDRs were considered adequate to validate our FFQ. It is worth mentioning that Mouratidou et al., (2006) [46], Mejía-Rodríguez et al., (2012) [51], and Brunst et al., (2016) [65], also checked the validity of their FFQ against two 24HDRs, having as testing period one month in pregnancy.

As already mentioned, the FFQ and the 24HDRs were interviewer-administered. In more detail, the FFQ and the first 24HDR were completed prior the antenatal appointment via a face-to-face interview.

For the completion of the 24HDR, participants were asked to recall in a meal-sequence base all foods and beverages consumed during the previous day. Participants were assisted in their estimation of portion sizes with commonly used household measures. Additional questions were documented regarding the preparation and serving methods. At the end of each interview, the participant’s responses were summarized by the interviewer in chronological order. Participants were, also, asked to declare whether the recalled day reflected their usual dietary habits or not, due to specific circumstances, e.g., sickness or celebration.

Approximately a week later, a second unannounced 24HDR was completed via the phone. For each participant, all interviews were conducted by the same interviewer in order to minimize inter-rater bias.

#### 2.2.5. Exclusion Criteria

The exclusion process was conducted in two phases. At a first level, 17 women were not included in the study for the following reasons: (a) 5 were diagnosed with pathologies, such as diabetes and hypertension; requiring a specific diet and (b) 12 could not provide appropriate dietary information. Among the latter, 7 (out of 12) were lost to follow up at the second interview, 3 (out of 12) were removed due to the inconsistency of answers, as evaluated by the cross-check and summary questions, and 2 (out of 12) were excluded since their culinary choices did not correspond to food items included in the FFQ’s food list. Furthermore, at a second level and once nutrient estimates were derived, 4 participants with biological improbable intakes (caloric intake greater than 3760 kcal per day) were excluded. This cut-off point was established taking into consideration the Willett’s arbitrary allowable range for women (500 to 3500 kcal per day) [12], as well as the guidelines of European Food Safety Authority (EFSA) (2013) [66]. The latter suggests an additional daily energy intake of 260 kcal per day, during the second trimester [66]. Application of all the above criteria resulted in a total of 179 women who were finally included in the validation study.

#### 2.2.6. Statistical Analyses

All statistical analyses were performed with SPSS v.15.0 (SPSS Inc., Chicago, IL, USA). The significance level in all hypothesis testing procedures was predetermined at *p* ≤ 0.05. Demographic/anthropometric characteristics and lifestyle factors of pregnant women are presented as means (SD-standard deviation) for quantitative data. For qualitative data, the number of subjects and the corresponding percentages (%) are given.

Nutrient intakes estimated by the FFQ and the average of the two 24HDRs are presented as mean (SD), median, minimum and maximum values, 25 and 75 percentiles. Measures of skewness and kurtosis, as well as the Kolmogorov-Smirnov test were used to check for normality of the data. Paired-samples *t* test and Wilcoxon rank-test were conducted to test the differences between nutrient intakes estimated by the two methods (FFQ vs. 24HDRs). Effect sizes for significant comparisons were estimated by Cohen’s d [67]; computations were done by means of the Microsoft excel. For Cohen’s d, an effect size below 0.3 may be interpreted as “small”, around 0.5 as “moderate”, and 0.8 or more as “large” effect [67]. The agreement between the two methods was examined as proposed by Bland and Altman (1986) [68].

The correlation between the two dietary methods was studied using Pearson and Spearman coefficients, for normally and not normally distributed variables, respectively.

In all non-parametric statistical hypothesis testing procedures (including also the significance test for Pearson’s correlation coefficient) the observed significance level (*p*-value) was computed with the Monte-Carlo simulation method based on 10,000 randomization circles [69]. This approach leads to valid inferential conclusions, even in cases where the methodological presuppositions of the corresponding hypothesis testing procedures are not met. 

Ranking agreement was examined by classification of nutrient intakes into same or adjacent quartile (correct classification) and into extreme-opposite quartile (gross misclassification) by the FFQ and the averaged 24HDRs. In order to compare quartiles of intake for each nutrient, the Cohen’s weighted kappa values were calculated [70], using a syntax code in SPSS. Finally, the agreement between FFQ and the averaged 24HDRs was further assessed by the intraclass correlation coefficient (ICC, model: two way mixed and absolute agreement). The values obtained for the Cohen’s weighted kappa and ICC were interpreted according to the cut-off points proposed by Landis and Koch (1977) [71]; thus, values of less than 0.21 indicate poor agreement, 0.21 to 0.40 fair agreement, 0.41 to 0.60 moderate agreement, 0.61 to 0.80 substantial agreement, and greater than 0.80 almost perfect agreement.

It must be noted that the norms for weighted kappa, ICC, as well as the Cohen’ s d, are simple conventions and their meaning and evaluation are based on the scientific field in which they are computed and used; nonetheless, they are very useful for relative comparisons within the same study, or between similar studies.

## 3. Results

The final culture-specific FFQ was composed of a matrix of 221 foods grouped into 22 categories. Regarding portion sizes, a consensus of opinion among the 50 women participating in the FFQ development, was introduced. Furthermore, the “precise frequency” was adopted, due to the higher accuracy of the information obtained. 

Demographic/anthropometric characteristics and lifestyle factors of the 179 pregnant women, who participated in the study, are summarized in Table 2. Furthermore, the MDS of the participants was relatively low—ranging from 18 to 41 (out of 50) (mean ± SD:31 ± 4). At this point, it should be mentioned that, since our study population involved pregnant women, it was considered obligatory to modify this score [7] in line with the assumptions made by Chatzi et al., (2012) [29]. Therefore, we considered dairy products to contain health effective dietary components and we did not include alcohol consumption in the MDS. Applying these modifications, the MDS ranged from 0 to 50. The diet transition to more westernized dietary patterns was further mirrored in the relatively high dietary GI ranging from 52.2 to 86.8 (mean ± SD:72.1 ± 7.1) and probably ensuing from the increased consumption of processed foods, due to the globalization of dietary habits.

For the validation study, descriptive statistics for energy, macro- and micronutrient intakes, as assessed by the FFQ and the averaged 24HDRs, are presented in Table 3, while the mean differences of these dietary parameters are given in Table 4.

Energy, plant protein, carbohydrates, fiber, and monounsaturated fatty acids (MUFA) mean intakes, as estimated by the FFQ, were significantly higher than the intakes estimated by the averaged 24HDRs. In contrast, the mean intake of animal protein, as well as the percentage of energy derived from total and animal protein, was lower. Concerning vitamins and elements, the estimated mean intake of 10 micronutrients by the FFQ was significantly higher, while that of sodium was significantly lower, when compared with the averaged 24HDRs (Table 4). However, it is of interest to note that statistically significant mean differences between the FFQ and the averaged 24HDRs were not important in terms of nutritional information. Indeed, effect sizes (Cohen’s d) ranged from 0.15 for vitamin B_6_ to 0.42 for magnesium and, for the majority of nutrients, were below 0.3 (Table 4). According to Cohen’s rule [67], an effect size of 0.2 to 0.3 can be regarded as “small”.

**Figure 2:**
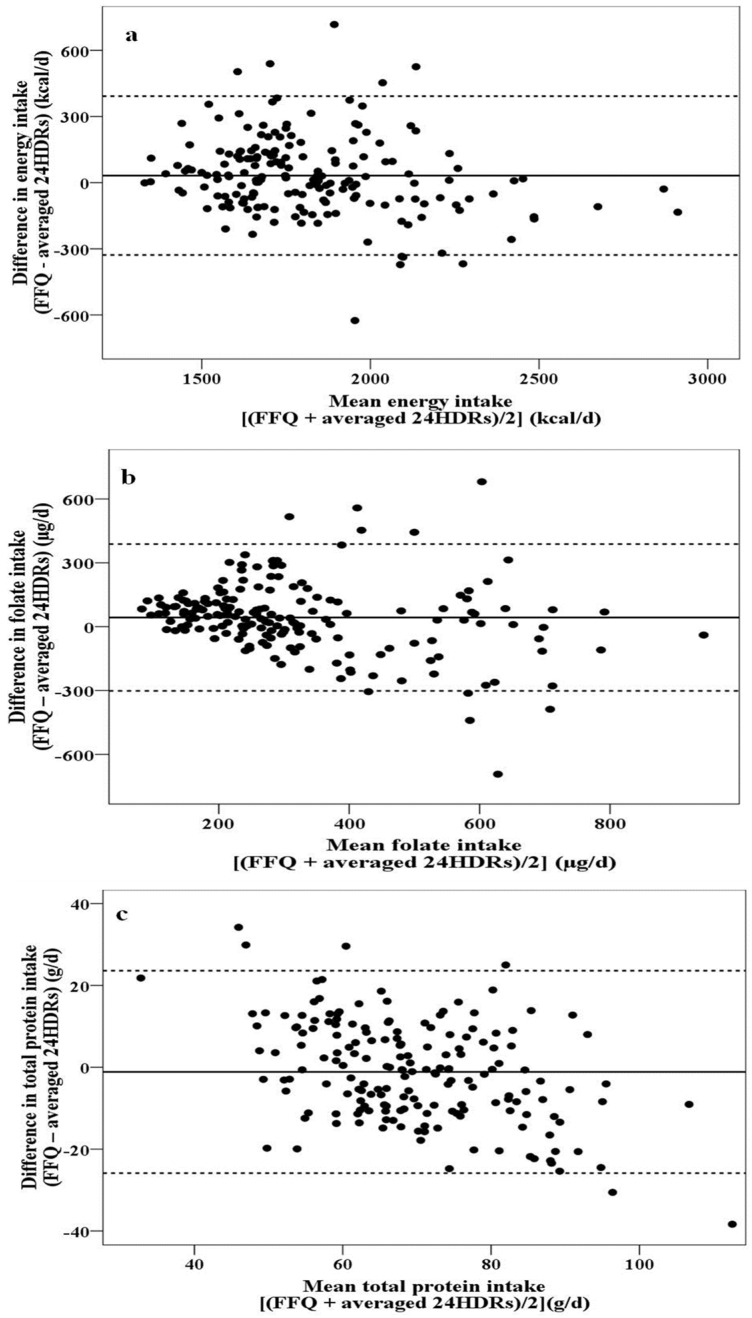
Differences in estimated (**a**) energy; (**b**) folate; and (**c**) protein intake calculated using the food-frequency questionnaire (FFQ) and the averaged 24-h dietary recalls (24HDRs).

The lower and upper limits of agreement—resulting from the Bland-Altman analysis—for all nutrients are, also, given in Table 4. The limits of agreement were nutritionally acceptable and ranged from positive to negative values, implying that the dietary intake was both over- and underestimated by the FFQ compared to the 24HDRs, respectively. Generally, three different patterns were observed, when the findings of Bland-Altman were plotted:

(a) no relationship between differences and mean values were observed (Figure 2a), i.e., the agreement between the two methods is of the same magnitude irrespective of intake quantity (energy, carbohydrates, total lipids, MUFA, saturated fatty acids (SFA), cholesterol, percentage of energy derived from carbohydrates, percentage of energy derived from total lipids, percentage of energy derived from MUFA, percentage of energy derived from SFA, calcium, phosphorus, magnesium, and potassium),
(b) a more scattered plot with increasing mean values was obtained (Figure 2b), meaning that the differences between the two methods are greater at the highest intakes (fiber, polyunsaturated fatty acids (PUFA), percentage of energy derived from PUFA, thiamine, niacin, pantothenic acid, vitamin B_6_, folate, vitamin B_12_, vitamin C, vitamin E, iron, and zinc), and
(c) increasing negative differences with increasing mean values were noted (Figure 2c), meaning that, compared with the FFQ, the 24HDRs overestimate the intake as the intake quantity increases (total protein, plant protein, animal protein, percentage of energy derived from total protein, percentage of energy derived from plant protein, percentage of energy derived from animal protein, vitamin A, and sodium).

Figure 2a–c depicts representative plots of the three different pattern modes. In all cases, more than 95% of the points fell within the limits of agreement.

As shown in Table 5, positive statistically significant correlations were noted for all nutrients, between the FFQ and the averaged 24HDRs, ranging from 0.35 for cholesterol to 0.77 for SFA. Classification between the two methods, weighted kappa values, and ICC are, also, presented in Table 5. The classification into quartiles showed that the overall proportion of pregnant women was classified into the same or adjacent quartile, when ranked by the FFQ and the averaged 24HDRs. Particular, the proportion of women classified correctly ranged from 73.2% for the percentage of energy derived from animal protein and cholesterol to 92.7% for energy. Gross misclassification was low for all nutrients; the highest percentage was recorded for MUFA, as well as the percentage of energy derived from plant protein, MUFA, and PUFA (6.1%), and the lowest for total protein (0.0%) (Table 5). Weighted kappa values were between 0.31 (MUFA) and 0.78 (percentage of energy derived from SFA), while ICC ranged between 0.49 (percentage of energy derived from plant protein) and 0.89 (energy) (Table 5). In addition, the fact that, for the majority of nutrients, the weighted kappa and ICC values showed moderate to substantial agreement denotes the ability of our FFQ to sufficiently evaluate energy, macro- and micronutrient intakes.

## 4. Discussion

The present study describes the development of a Mediterranean oriented culture-specific semi-quantitative FFQ, as well as the evaluation of its relative validity in measuring energy and nutrient intakes in a population of pregnant women.

### 4.1. Development of the Culture-Specific FFQ

In an attempt to capture the dietary habits of culturally diverse population groups, many culture-specific FFQs have been developed [11,72,73,74,75,76,77,78,79,80]. According to Teufel (1997) [20] and Sharma (2011) [21], there are some main steps for designing a culture-specific FFQ, including the development of a complete and precise food list, the determination of culture-specific food groups, and the definition of culturally appropriate portion sizes. Therefore, in line with other studies [11,21,72,75,77,81], our methodological framework was based on the aforementioned guidelines.

It is important to highlight that the challenge confronted in the present study was to merge, in our FFQ, food items and practices reflecting cultural Mediterranean preferences with other food choices ensuing from the reported diet transition towards more westernized dietary patterns [5,6,7,8,10,11]. This approach was a prerequisite to ensure a reasonable degree of accuracy of the FFQ, as diet is an array of options shaped by cultural, ethnic, and personal preferences, as well as current trends, food cost, and availability.

### 4.2. FFQ Validation

The classical statistical approaches in validation studies adopted Pearson and Spearman correlation analyses, as well as paired-samples *t* test or non-parametric tests, such as the Wilcoxon rank-test [13,82]. However, correlation coefficients are formed to measure the relation, rather than the agreement between the two methods, i.e., FFQ and the reference method [13,82]. Furthermore, comparison of group means may lead to conclusions that are not meaningful in terms of nutritional information [82]. Thus, current scientific recommendations suggest the implementation of the classical approaches in conjunction with other statistical techniques, such as the Bland–Altman method, categorization into intake levels, and weighted kappa [13,82,83]. Our methodological strategy was to combine several statistical methods, in order to assess the relative validity of the developed FFQ.

Our finding that the FFQ slightly overestimates most of the nutrient intakes, at a group level, concurs with the observations of other researchers [11,24,32,46,47,56,57,63,84,85,86,87,88]. This usual tendency of FFQs to overestimate nutrient intakes may be attributed to the extended list of food items used [11,13,24,80,85,86]. In addition, the three different pattern modes, as ensued from the Bland-Altman analysis, have already been previously reported by Hjartåker et al. (2007) [89].

Our data indicate a relatively high correlation level between the FFQ and the reference method. According to Willett (1998) [12] and Cade et al., (2002) [13], values for correlation coefficients between 0.5 and 0.7 are considered to be the best attainable in dietary validation studies. The fact that, in our study, the correlation coefficients were above 0.5, for most of the nutrients (28 out of 36), may be attributed to the synergistic effect of the complete and precise food list, the method of administration—i.e., the personal interview—and the type of questions—i.e., open-ended [13]. Regarding categorization into intake levels, the quartile notation in the present study was good and our findings comparable to those of previous reports [11,32,52,63,84,90,91].

### 4.3. Limitations of the Study

For the validation of our FFQ, the 24HDR was selected as the reference method. This approach could be viewed as bearing inherent possible weakness, since both FFQ and 24HDR have similar measurement errors, i.e., memory bias [13,45,47,52]. However, the sample size of 179 women was large enough to counterbalance any plausible implications on the results. Furthermore, under the conditions of our study, the weighted dietary records would have rendered the evaluation of dietary intake more difficult for the following reasons: apart from being more expensive, they place a relative large respondent’s burden, since training is obligatory before participation in the survey [92]. It is also worth noting that the less familiar with the procedure the population is, the more complicate and time-consuming the training.

### 4.4. Strengths of the Study

A major strength of the present study was the extensive process adopted to develop a detailed food list and define the appropriate portion sizes and food groups, within a Mediterranean oriented culture-specific framework. Furthermore, our tool for converting participants’ responses into dietary data was designed to calculate, among others, the dietary GI and MDS, facilitating, thus, the assessment of adherence to the Mediterranean dietary pattern.

Regarding the validation stage, the strengths lie on the sample size and data collection phase [13,32,91]. First, the sample size of 179 individuals is above the usual number employed in other validation studies involving pregnant women [93]. Indeed, one of the five components of the scoring system [14] aiming to assess the quality of dietary intake in validation studies is the sample size; the quality criterion of 100 individuals is generally considered as appropriate in research efforts with similar methodology (24HDRs or dietary records as reference method). With respect to the data collection phase, the direct communication between the trained interviewer and the participants rendered the responses more spontaneous and, thus, more reliable. 

The statistical methodology applied constitutes a further strength of this study. In a recent review, Lombard et al., (2015) [83] reported that the number of statistical tests typically used in validation studies varies between one and three. However, we applied almost all acceptable methods, developing a methodological framework that, according to the literature [83], is sufficient to provide comprehensive insights into the various aspects of validity.

## 5. Conclusions

A Mediterranean oriented culture-specific semi-quantitative FFQ was developed for assessing energy and nutrient intakes and subsequently applied to a population of pregnant women to test its validity.

For FFQ development, the main challenge encountered was to take into account both food items and practices reflecting cultural Mediterranean preferences, as well as food choices resulting from diet transition to more westernized type patterns. Furthermore, the validity of the developed FFQ was proven using classical statistical approaches in conjunction with other statistical techniques, when applied to the target group.

In conclusion, our working framework using the developed FFQ to generate data of energy and nutrient intakes could serve as a dietary assessment tool for relevant nutritional studies in the Mediterranean region.

## Figures and Tables

**Table 1:** Steps followed for the calculation of the daily dietary intake.

***Step 1. Convert Frequency Consumption Data into Grams of Food per Day.***
For this purpose, any weekly or monthly frequency of consumption was converted into daily, by dividing with 7 or 30, respectively. The daily frequency of consumption by food was, then, multiplied by the appropriate portion size in grams.
***Step 2. Calculate Daily Nutrient Intake by Food.***
The daily nutrient intake by food was computed by multiplying the food intake (in grams) by the corresponding nutrient content per gram.
***Step 3. Calculate Daily Nutrient Intake by Food Group.***
Daily nutrient intake by food group was computed by summing the daily nutrient contribution of all food items belonging to this food category.
***Step 4. Calculate Total Daily Nutrient Intake.***
Total daily nutrient intake was computed by summing up the daily nutrient contributions of all food items.
***Step 5. Compute the Nutrient Density (Nutrient Intake per Energy Intake) by Food/Food Group and Overall.***
***Step 6. Compute Dietary GI and MDS.***
Dietary GI was calculated according to Hu et al., (2009) [37], using white bread as the standard reference. GI values of the different food items were derived from published international tables [38].
MDS was computed according to Panagiotakos et al., (2006) [7].

GI: Glycemic index; MDS: Mediterranean Diet Score.

**Table 2:** Demographic/anthropometric characteristics and lifestyle factors of the 179 pregnant women who participated in the present study.

**Characteristic**	**Mean (SD)**
Maternal age (year)	36.8 (4.1)
Pre-pregnancy BMI (kg/m^2^)	24.1 (4.8)
**Characteristic**	***n* (%)**
Pre-pregnancy BMI	
Underweight (BMI < 18.5 kg/m^2^)	7 (3.9)
Normal (BMI 18.5–24.9 kg/m^2^)	108 (60.3)
Overweight (BMI 25–29.9 kg/m^2^)	43 (24.0)
Obese (BMI ≥ 30 kg/m^2^)	21 (11.8)
Education	
9 years	10 (5.6)
12 years	69 (38.6)
>12 years	100 (55.8)
Working during pregnancy	
Yes	103 (57.5)
No	76 (42.5)
Smoking during pregnancy	
Yes	26 (14.5)
No	153 (85.5)
Physical activity level	
Low activity	147 (82.1)
Moderate activity	17 (9.5)
High activity	15 (8.4)

SD: standard deviation; BMI: body mass index.

**Table 3:** Daily energy, macro- and micronutrient intakes estimated by the food-frequency questionnaire (FFQ) and the averaged 24-h dietary recalls (24HDRs) (*n* = 179).

		Min	Q_25_	Median	Q_75_	Max	Mean (SD)	SE	Skewness	Kurtosis	K-S *p*
Energy (kcal)	**F**	1330	1645	1800	1980	2854	1838 (271)	20	0.9	1.4	0.12
	**R**	1294	1599	1738	1937	2977	1806 (310)	23	1.1	1.4	0.03
Total protein (g)	**F**	40	61	68	77	102	69 (11)	1	0.3	0.03	0.59
	**R**	22	60	69	79	132	70 (16)	1	0.4	0.9	0.38
Plant protein (g)	**F**	12	19	23	28	39	23 (5)	0.4	0.4	−0.2	0.51
	**R**	4	15	21	28	48	22 (8)	1	0.5	0.04	0.45
Animal protein (g)	**F**	24	39	44	53	79	45 (11)	1	0.4	0.4	0.28
	**R**	6	35	47	59	118	48 (18)	1	0.4	0.8	0.88
Carbohydrates (g)	**F**	103	151	172	194	326	174 (34)	3	0.9	2.2	0.12
	**R**	86	142	164	190	308	170 (41)	3	0.9	1.3	0.05
Fiber (g)	**F**	7	14	17	21	37	18 (5)	0.4	0.8	0.7	0.29
	**R**	4	11	15	20	40	17 (7)	1	0.9	0.5	0.05
Total lipids (g)	**F**	59	81	89	98	147	91 (15)	1	0.8	1.1	0.08
	**R**	46	78	88	99	152	89 (17)	1	0.7	1.5	0.27
MUFA (g)	**F**	28	40	44	49	73	45 (7)	1	0.9	1.9	0.15
	**R**	24	37	43	48	81	43 (9)	1	0.6	1.6	0.64
PUFA (g)	**F**	7	10	11	14	27	12 (3)	0.3	1.2	2	0.003
	**R**	4	9	11	14	35	12 (5)	0.4	1.6	3.7	<0.001
SFA (g)	**F**	14	23	28	31	51	28 (7)	1	0.7	1	0.29
	**R**	8	22	27	33	57	28 (8)	1	0.8	1.1	0.05
Cholesterol (mg)	**F**	75	186	219	262	437	229 (67)	5	0.8	1.1	0.15
	**R**	36	154	207	268	625	226 (102)	8	1	1.3	0.04
% Energy from total protein	**F**	10	14	15	16	22	15 (2)	0.1	0.2	1.1	0.41
	**R**	6	14	16	17	28	16 (3)	0.2	0.4	2.3	0.83
% Energy from plant protein	**F**	3	4	5	6	8	5 (1)	0.1	0.2	−0.1	0.54
	**R**	1	4	5	6	9	5 (2)	0.1	0.2	−0.5	0.91
% Energy from animal protein	**F**	4	9	10	11	16	10 (2)	0.2	0.2	0.2	0.26
	**R**	2	8	11	13	25	11 (4)	0.3	0.3	1.2	0.75
% Energy from carbohydrates	**F**	26	35	37	40	50	38 (4)	0.3	0.4	0.4	0.25
	**R**	20	34	37	41	62	37 (6)	0.4	0.4	2.1	0.52
% Energy from total lipids	**F**	33	42	45	48	53	45 (4)	0.3	−0.4	0.02	0.78
	**R**	30	42	45	48	55	45 (5)	0.4	−0.4	0.02	0.64
% Energy from MUFA	**F**	16	20	22	24	29	22 (2)	0.2	0.2	−0.2	0.94
	**R**	12	19	21	24	32	22 (4)	0.3	0.2	−0.2	0.87
% Energy from PUFA	**F**	4	5	6	7	15	6 (1)	0.1	2.5	11	0.009
	**R**	0	4	5	7	14	6 (2)	0.2	0.8	2.1	0.001
% Energy from SFA	**F**	8	12	13	15	19	14 (2)	0.2	0.1	0.1	0.78
	**R**	5	12	14	16	22	14 (3)	0.2	−0.1	−0.1	0.96
Thiamin (mg)	**F**	0.6	1.2	1.4	1.8	3.5	1.6 (0.6)	0.04	1.1	1	0.03
	**R**	0.3	1	1.4	2.1	3.8	1.6 (0.7)	0.05	0.6	−0.4	0.01
Riboflavin (mg)	**F**	0.9	1.7	2.1	2.6	4.6	2.2 (0.8)	0.06	0.8	0.2	0.11
	**R**	0.6	1.4	1.8	2.5	4.7	2 (0.9)	0.06	0.9	0.3	0.004
Niacin (mg)	**F**	7	14	17	22	44	19 (7)	1	1.1	0.7	0.01
	**R**	2	13	18	26	49	19 (9)	1	0.7	0.1	0.1
Pantothenic acid (mg)	**F**	3	4	5	9	22	7 (4)	0.3	1.3	0.9	<0.001
	**R**	1	4	5	6	20	6 (4)	0.3	1.4	0.5	<0.001
Vitamin B_6_ (mg)	**F**	0.9	1.4	1.7	2.3	5.6	2 (0.8)	0.06	1.4	2.1	0.001
	**R**	0.4	1.3	1.6	2.1	4.4	1.9 (0.9)	0.07	1	0.2	<0.001
Folate (μg)	**F**	113	225	292	426	944	346 (171)	12	1.2	1.1	0.001
	**R**	30	140	244	409	975	303 (210)	16	1.1	0.7	0.001
Vitamin B_12_ (μg)	**F**	1.6	3	4.5	6.3	14.3	5.1 (2.6)	0.2	1.1	0.5	0.01
	**R**	0.2	2.4	3.8	7.1	17.7	4.8 (3.2)	0.2	1.1	0.7	<0.001
Vitamin C (mg)	**F**	23	80	117	167	419	134 (71)	5	1.2	1.5	0.017
	**R**	3	43	95	153	421	110 (87)	7	1.1	0.8	0.03
Vitamin A (RAE)	**F**	205	410	499	606	1164	511 (155)	12	0.8	1.5	0.38
	**R**	79	281	420	704	1642	503 (290)	22	1	0.7	0.007
Vitamin E (mg)	**F**	5	9	12	17	37	14 (6)	0.5	1.3	1.4	0.003
	**R**	3	7	10	15	32	12 (7)	0.5	1.1	0.3	<0.001
Calcium (mg)	**F**	454	857	1011	1221	2035	1044 (279)	21	0.6	0.7	0.35
	**R**	304	728	957	1177	2468	979 (336)	25	0.7	1.7	0.87
Phosphorus (mg)	**F**	716	1049	1221	1383	2086	1223 (250)	19	0.5	0.4	0.57
	**R**	426	949	1116	1381	2333	1172 (314)	23	0.7	0.9	0.12
Magnesium (mg)	**F**	136	207	243	279	425	248 (53)	4	0.5	0.1	0.51
	**R**	98	183	213	265	532	226 (66)	5	1.1	2.4	0.05
Potassium (mg)	**F**	1360	2077	2389	2692	4191	2428 (503)	38	0.6	0.7	0.41
	**R**	633	1770	2262	2749	4217	2276 (685)	51	0.3	−0.1	0.61
Sodium (mg)	**F**	1242	1790	2027	2323	3557	2094 (468)	35	0.7	0.6	0.16
	**R**	731	1719	2078	2690	5014	2264 (816)	61	0.9	0.8	0.003
Iron (mg)	**F**	6	9	12	17	39	14 (7)	1	1.3	1.2	<0.001
	**R**	3	8	10	17	37	13 (8)	1	1.1	0.1	<0.001
Zinc (mg)	**F**	5	9	11	15	34	13 (6)	0.4	1.3	0.9	<0.001
	**R**	3	8	11	15	32	13 (7)	0.5	1.2	0.4	<0.001

F: food-frequency questionnaire; R: averaged 24-h dietary recalls; K-S: Kolmogorov-Smirnov test for normality; MUFA: Monounsaturated fatty acids; PUFA: Polyunsaturated fatty acids; SFA: Saturated fatty acids. Macro- and most micronutrients are given as whole numbers, in order to simplify the presentation of the data.

**Table 4:** Test of difference between the food-frequency questionnaire (FFQ) and the averaged 24-h dietary recalls (24HDRs) (*n* = 179).

		Mean Difference * (SD)	Skewness	*p*	Kurtosis	K-S *p*	Cohen’s d	Lower LOA	Upper LOA
Macronutrients with no statistically significant difference	Total protein (g)	−1.13 (12.37)	0.02	0.22	0.00	0.93		−25.87	23.60
	Total lipids (g)	1.67 (12.17)	0.13	0.069	0.52	0.98		−22.68	26.01
	PUFA (g)	0.09 (3.84)	−0.30	0.38	1.54	0.18		−7.59	7.77
	SFA (g)	0.16 (5.39)	−0.64	0.70	0.83	0.64		−10.62	10.94
	Cholesterol (mg)	3.15 (98.09)	−0.88	0.67	1.73	0.10		−193.04	199.34
	% Energy from plant protein	0.21 (1.62)	−0.14	0.077	−0.25	0.64		−3.03	3.46
	% Energy from carbohydrates	0.35 (4.89)	−0.05	0.35	0.69	0.74		−9.43	10.12
	% Energy from total lipids	−0.02 (4.51)	−0.10	0.94	−0.05	0.92		−9.04	9.00
	% Energy from MUFA	0.34 (3.61)	−0.19	0.21	0.55	0.31		−6.88	7.56
	% Energy from PUFA	0.20 (2.02)	0.29	0.18	1.49	0.02		−3.83	4.24
	% Energy from SFA	−0.13 (2.25)	−0.16	0.44	−0.39	0.94		−4.64	4.37
Macronutrients significantly higher in FFQ	Energy (kcal)	32.10 (180.01) †	0.35	0.018	1.85	0.25	0.16	−327.92	392.13
	Plant Protein (g)	1.45 (7.31) †	−0.37	0.009	−0.06	0.70	0.21	−13.16	16.06
	Carbohydrates (g)	4.48 (28.45) †	−0.39	0.036	1.04	0.48	0.16	−52.42	61.39
	Fiber (g)	1.52 (5.96) †	−0.64	0.001	2.05	0.50	0.27	−10.40	13.44
	MUFA (g)	1.66 (7.81) †	0.20	0.005	0.36	0.91	0.22	−13.96	17.28
Macronutrients significantly lower in FFQ	Animal Protein (g)	−2.60 (15.59) †	0.00	0.027	0.19	0.96	−0.18	−33.77	28.58
	%Energy from total protein	−0.49 (2.39) †	−0.02	0.006	−0.18	0.96	−0.22	−5.28	4.30
	%Energy from animal protein	−0.71 (3.27) †	0.00	0.004	0.02	0.78	−0.24	−7.24	5.82
Micronutrients with no statistically significant difference	Thiamin (mg)	−0.01 (0.59)	0.34	0.90	1.27	0.55		−1.19	1.18
	Niacin (mg)	−0.47 (7.77)	0.83	0.42	3.44	0.19		−16.01	15.08
	Vitamin B_12_ (μg)	0.37 (2.75)	0.36	0.070	2.66	0.38		−5.13	5.88
	Vitamin A (RAE)	7.42 (264.39)	−1.07	0.71	2.10	0.09		−521.36	536.19
	Iron (mg)	0.71 (6.13)	0.61	0.14	2.68	<0.001		−11.55	12.97
	Zinc (mg)	0.25 (5.64)	0.45	0.56	3.00	0.11		−11.03	11.52
Micronutrients significantly higher in FFQ	Riboflavin (mg)	0.24 (0.66) †	0.35	<0.001	1.88	0.60	0.36	−1.08	1.56
	Pantothenic acid (mg)	0.92 (3.50) §	0.48	<0.001	3.83	0.01	0.23	−6.08	7.92
	Vitamin B_6_ (mg)	0.12 (0.72) §	0.61	0.029	3.09	0.02	0.15	−1.32	1.56
	Folate (μg)	43.23 (172.46) †	−0.09	0.001	2.92	0.05	0.25	−301.69	388.15
	Vitamin C (mg)	23.65 (67.43) †	−0.20	<0.001	1.72	0.77	0.34	−111.21	158.51
	Vitamin E (mg)	1.57 (5.05) †	0.30	<0.001	1.19	0.21	0.28	−8.52	11.67
	Calcium (mg)	64.77 (269.50) †	−0.34	0.002	1.06	0.16	0.24	−474.24	603.77
	Phosphorus (mg)	50.39 (228.73) †	−0.29	0.004	0.13	0.84	0.23	−407.08	507.86
	Magnesium (mg)	21.39 (47.48) †	0.01	<0.001	−0.22	0.90	0.42	−73.57	116.35
	Potassium (mg)	151.23 (519.84) †	−0.02	<0.001	−0.08	0.93	0.31	−888.45	1190.91
Micronutrients significantly lower in FFQ	Sodium (mg)	−169.46 (654.62) †	−0.97	0.001	1.68	0.11	−0.30	−1478.70	1139.77

* FFQ—averaged 24HDRs; † Paired-samples *t* test; § Wilcoxon rank-test; K-S: Kolmogorov-Smirnov test for normality; LOA: Limit of agreement (Bland-Altman method).

**Table 5:** Correlation coefficients (by Pearson and Spearman), weighted kappa, and intraclass correlation coefficients (ICC) between daily nutrient intakes. Cross classification of pregnant women by quartiles of nutrient intakes, as estimated by the food-frequency questionnaire (FFQ) and the averaged 24-h dietary recalls (24HDRs) (*n* = 179).

	Correlation Coefficient *	Weighted Kappa †	ICC †	Correctly %	Grossly %
Energy (kcal)	0.75 §	0.71	0.89	92.7	1.1
Total protein (g)	0.66 ‡	0.63	0.76	89.9	0.0
Plant protein (g)	0.52 ‡	0.49	0.63	81.6	2.2
Animal protein (g)	0.50 ‡	0.44	0.60	78.2	2.8
Carbohydrates (g)	0.73 ‡	0.60	0.83	86.6	1.1
Fiber (g)	0.61 ‡	0.56	0.72	84.9	1.7
Total lipids (g)	0.72 ‡	0.41	0.83	88.3	2.2
MUFA (g)	0.55 ‡	0.31	0.69	78.8	6.1
PUFA (g)	0.54 §	0.50	0.73	83.2	3.9
SFA (g)	0.77 ‡	0.58	0.86	90.5	0.6
Cholesterol (mg)	0.35 §	0.32	0.53	73.2	3.9
% Energy from total protein	0.55 ‡	0.41	0.65	77.6	3.9
% Energy from plant protein	0.39 ‡	0.37	0.49	78.8	6.1
% Energy from animal protein	0.45 ‡	0.34	0.55	73.2	3.9
% Energy from carbohydrates	0.52 ‡	0.43	0.66	78.2	3.3
% Energy from total lipids	0.45 ‡	0.61	0.61	77.1	3.9
% Energy from MUFA	0.38 ‡	0.51	0.51	76.0	6.1
% Energy from PUFA	0.41 §	0.37	0.63	78.2	6.1
% Energy from SFA	0.66 ‡	0.78	0.78	86.6	2.8
Thiamin (mg)	0.61 §	0.59	0.74	86.6	2.2
Riboflavin (mg)	0.65 §	0.58	0.78	87.1	2.2
Niacin (mg)	0.56 §	0.55	0.72	88.3	3.9
Pantothenic acid (mg)	0.56 §	0.49	0.78	83.2	3.3
Vitamin B_6_ (mg)	0.56 §	0.52	0.71	82.7	3.3
Folate (μg)	0.59 §	0.55	0.74	84.4	2.8
Vitamin B_12_ (μg)	0.51 §	0.51	0.71	83.2	2.8
Vitamin C (mg)	0.62 §	0.61	0.76	86.0	1.1
Vitamin A (RAE)	0.46 §	0.40	0.52	78.8	3.3
Vitamin E (mg)	0.63 §	0.58	0.81	86.6	1.7
Calcium (mg)	0.63 ‡	0.55	0.76	85.5	2.8
Phosphorus (mg)	0.69 ‡	0.66	0.80	89.9	1.1
Magnesium (mg)	0.63 §	0.56	0.78	85.4	1.7
Potassium (mg)	0.66 ‡	0.54	0.76	86.0	2.2
Sodium (mg)	0.60 §	0.57	0.67	86.0	2.2
Iron (mg)	0.61 §	0.56	0.78	85.5	2.8
Zinc (mg)	0.52 §	0.47	0.76	79.3	2.8

* For all correlation coefficients *p* < 0.001; † Weighted kappa was calculated for categorized data and intraclass correlation coefficient was calculated for raw data; § Spearman rank correlation; ‡ Pearson correlation.
